# Identification of miRNA biomarkers for breast cancer by combining ensemble regularized multinomial logistic regression and Cox regression

**DOI:** 10.1186/s12859-022-04982-7

**Published:** 2022-10-18

**Authors:** Juntao Li, Hongmei Zhang, Fugen Gao

**Affiliations:** College of Mathematics and Information Science, Henan Normal University, Xinxiang, China

**Keywords:** Breast cancer, Feature selection, MicroRNAs, Biomarkers

## Abstract

**Background:**

Breast cancer is one of the most common cancers in women. It is necessary to classify breast cancer subtypes because different subtypes need specific treatment. Identifying biomarkers and classifying breast cancer subtypes is essential for developing appropriate treatment methods for patients. MiRNAs can be easily detected in tumor biopsy and play an inhibitory or promoting role in breast cancer, which are considered promising biomarkers for distinguishing subtypes.

**Results:**

A new method combing ensemble regularized multinomial logistic regression and Cox regression was proposed for identifying miRNA biomarkers in breast cancer. After adopting stratified sampling and bootstrap sampling, the most suitable sample subset for miRNA feature screening was determined via ensemble 100 regularized multinomial logistic regression models. 124 miRNAs that participated in the classification of at least 3 subtypes and appeared at least 50 times in 100 integrations were screened as features. 22 miRNAs from the proposed feature set were further identified as the biomarkers for breast cancer by using Cox regression based on survival analysis. The accuracy of 5 methods on the proposed feature set was significantly higher than on the other two feature sets. The results of 7 biological analyses illustrated the rationality of the identified biomarkers.

**Conclusions:**

The screened features can better distinguish breast cancer subtypes. Notably, the genes and proteins related to the proposed 22 miRNAs were considered oncogenes or inhibitors of breast cancer. 9 of the 22 miRNAs have been proved to be markers of breast cancer. Therefore, our results can be considered in future related research.

## Background

Breast cancer is a disease that affects women’s health [1]. The statistical results of the American Cancer Association on female breast cancer in the United States show that the incidence rate of breast cancer increased by 0.3% per year from 2012 to 2016 [2]. In addition, breast cancer was predicted to be the most common cancer in the United States in 2022 through open source data [3]. Fortunately, with the development of medical technology, the mortality rate of breast cancer continues to decline [2]. The therapeutic effect will be further improved through subtype specific-treatment. Breast cancer is commonly categorized into four main subtypes: Luminal A (LA), Luminal B (LB), HER2-Enriched (H2), and Basal-Like (BL) [4]. LA is the most common subtype of breast cancer, which accounted for 64% in all white patients and 48% in all African Americans patients in the study of [5]. Generally speaking, the prognosis of luminal subtypes is good, but LB is significantly worse than LA [6]. The prognosis of H2 and BL is poor. The BL tumor is larger than other subtypes and grows faster, which is the worst prognosis [5, 6]. Without losing generality, the normal sample can be regarded as the fifth subtype, i.e., control subtype.

More and more evidence shows that there are biological differences between subtypes of breast cancer [6, 7]. The mortality rate of 4 subtypes varies with time, and the response to specific treatment is different [7]. For example, LA can be adequately treated by endocrine therapy, while LB can be treated by a combination of chemotherapy and hormone therapy [6]. At present, many machine learning methods have been successfully applied to distinguish breast cancer patients from normal people, such as hierarchical clustering, random forest (RF), and Light Gradient Boosting Machine [8–10]. However, the key to improving the survival rate is to accurately judge the subtype of patients and provide appropriate treatment. Recent studies have shown that expression values of miRNA differ among the intrinsic subtypes of breast cancer and have great potential in diagnosing and treating breast cancer [11, 12].

MicroRNAs (miRNAs) are a 21-25 long class of small non-protein coding RNA that regulate an estimated 30% of all human genes and play an inhibitory or promoting role in cancer [12]. MiRNAs are considered promising breast cancer biomarkers because they can be easily detected in tumor biopsy [13]. Although data availability continues to increase, not all miRNAs are available in every study. Therefore, it is meaningful to find a small miRNA subset as feature set to classify breast cancer subtypes [14–16]. Lopez-Rincon et al. selected 100 features by integrating the results of 3 classification tree-based and 5 linear model-based machine learning methods [14]. Rehman et al. screened features and ranked importance through Information Gain, Chi-Squared, and Lasso [15]. Sarkar et al. believed that the features selected simultaneously by eight feature selection methods based on mutual information were important [16].

**Fig. 1:**
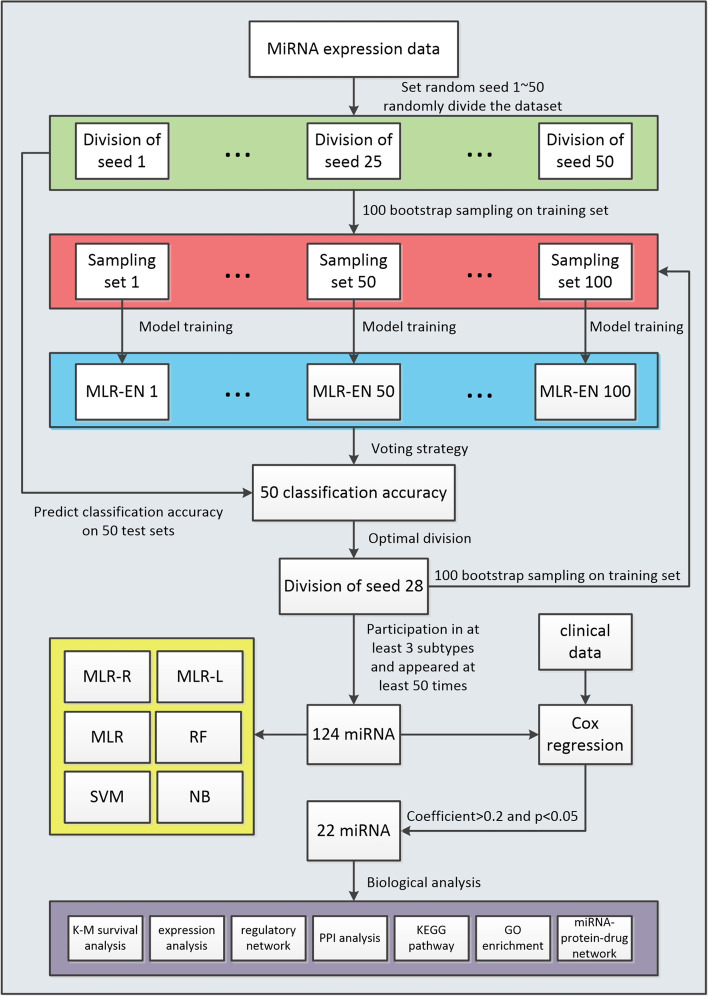
Overview of identifying miRNAs biomarkers

As successful feature selection methods based on ensemble learning, different classifiers on the same dataset are integrated to improve the performance of feature selection in [14, 16]. An alternative ensemble learning method is to integrate the same classifier (with different parameters) on different datasets. Motivated by this idea, we proposed a new identification method of miRNA biomarkers for breast cancer by combining ensemble regularized multinomial logistic regression and Cox regression. An overview of the method was presented in Fig 1.

Different from [14, 16], we adopted stratified sampling and bootstrap sampling to ensemble 100 multinomial logistic regression models with elastic net penalty (MLR-EN) in order to determine the most suitable sample subset for feature screening. Based on this, 124 miRNA were screened as features. In order to verify that the proposed feature set is not only applicable to a specific classifier, 6 machine learning methods were implemented, including multinomial logistic regression(MLR), multinomial logistic regression with ridge regression penalty (MLR-R), multinomial logistic regression with lasso penalty (MLR-L), RF, support vector machine (SVM) and naive Bayes (NB). Further, we identified 22 miRNAs as biomarkers through Cox regression based on survival analysis. The results of 7 biological analyses illustrate the rationality of the identified biomarkers.

## Results

### Feature screening

In order to avoid the influence of data division on feature selection, we first determined the sample subset that was most suitable for miRNA feature screening. To this end, we set up a random seed, and stratified sampling the miRNA-seq dataset containing 5 subtypes and obtained 80% (185) samples containing the total samples as the training set $Q_{train}$ and 20% (46) samples as the test set $Q_{test}$. Using this sampling method, 80% of each subtype was randomly selected to form the training set $Q_{train}$, i.e., 69 LA, 31 LB, 19 H2, 33 BL, and 33 control subtypes. Further, we conducted bootstrap sampling on the training set of each subtype and constructed 100 MLR-EN models. Following the idea on parameter selection in [17, 18], we specified a sequence value $\alpha = \{0.05, 0.2, 0.4, 0.6, 0.8, 0.95\}$ in advance, determined the parameter $\lambda$ for each prespecified $\alpha$ via the 10 fold cross-validation, and then determined the optimal parameter pair $(\alpha _{0}, \lambda _{0})$ for each MLR-EN model. Subsequently, we ensembled 100 MLR-EN models and predicted 5 subtypes on the test set $Q_{test}$ (17 LA, 8 LB, 5 H2, 8 BL, and 8 control subtypes) by voting strategy (see Algorithm 1 in the Methods). The above process was repeated 50 times. Both prediction accuracy and cross entropy loss [19] were used to evaluate the performance of multi-classification, and then the optimal data division was determined. In the calculation of cross entropy loss, we calculated the ratio of the number of votes obtained by the specified subtype to 100, and then took it as the prediction probability of belonging to the specified subtype (0.001 was adopted for making subsequent logarithmic operations meaningful if the obtained probability is 0). After calculation, the highest prediction accuracy of 91.30% and the lowest cross entropy loss of 0.4777 in 50 experiments were obtained on the test set when the random seed was set to 28. Results of accuracy and cross entropy loss for 50 experiments were shown in Additional file 1: Table S1. The training set $Q_{train}$ corresponding to random seed 28 was considered the most suitable sample subset for miRNA feature screening.

**Fig. 2:**
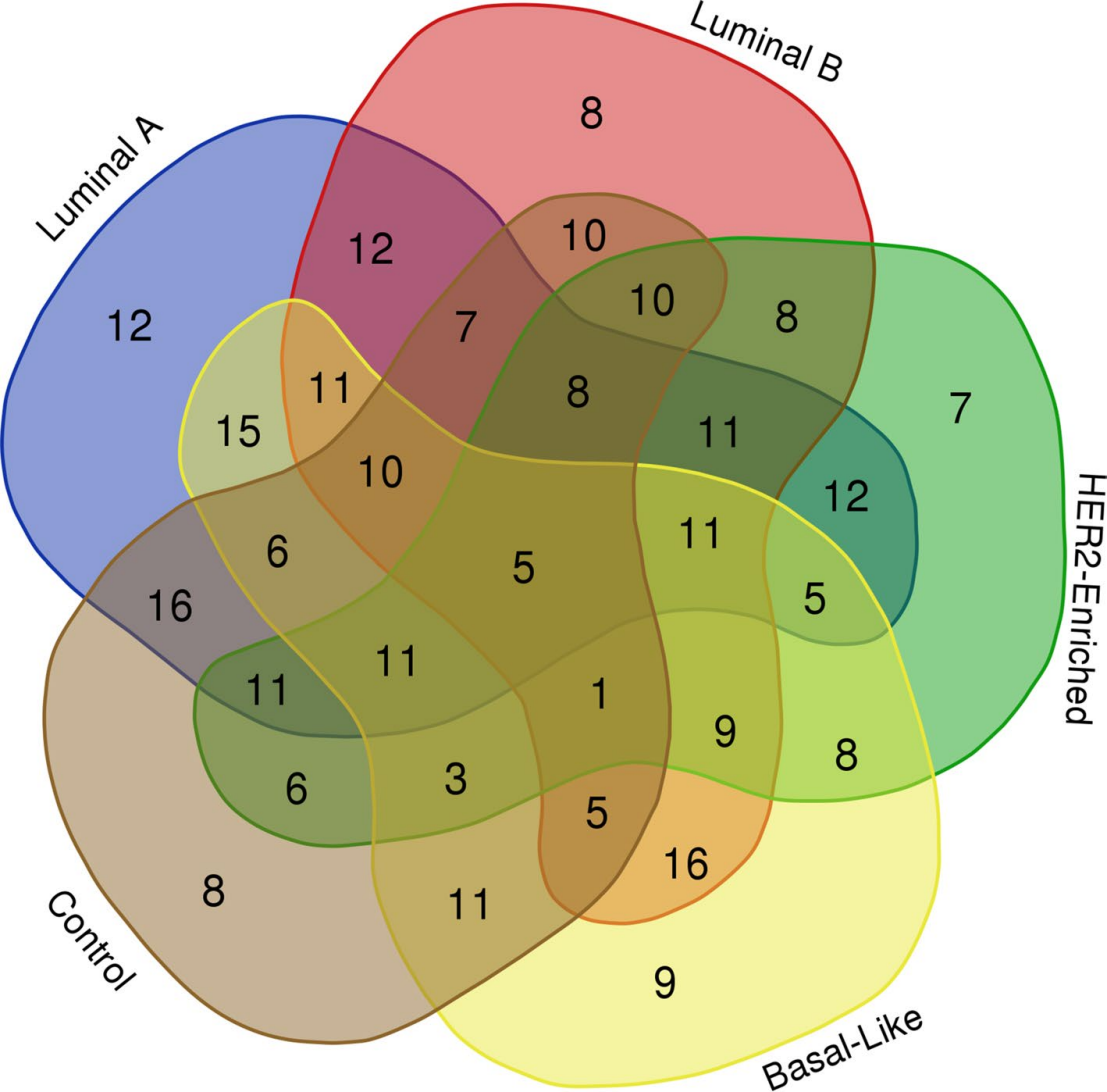
Number of miRNAs that participated in classification of each subtype

The number of miRNAs that participated in the classification of each subtype was shown in Fig. 2. According to the rationale of the voting strategy, only miRNAs that appeared more than 50 times in the 100 MLR-EN models on the division of seed 28 were reserved. A few miRNAs may participate in different subtypes and play different regulatory roles. Especially, miRNAs that participated in at least half of the subtypes were considered very important. Following this idea, 124 miRNAs that participated in at least 3 subtypes were further selected as features, and the specific names were shown in Additional file 1: Table S2.

MLR-R was performed to evaluate the performance of 5 feature sets participating in the different number of subtypes. Set random seeds 1–100 to randomly divide 80% of the samples as the training set and the remaining 20% as the test set 100 times. The average classification accuracy (ACA) and variance (Var) of MLR-R on the test set in 100 data division experiments were shown in Table 1. MLR-R achieved the highest ACA of 79.41% (95% confidence interval of 0.7839–0.8043) on the proposed feature set.

**Table 1:** The ACA and Var of MLR-R on 5 feature sets

No. of participating subtypes	MiRNA size	ACA(Var)
At least 1	282	0.7385 (0.0019)
At least 2	238	0.7417 (0.0015)
At least 3	124	**0.7941** (0.0026)
At least 4	46	0.7733 (0.0026)
At least 5	5	0.5965 (0.0029)

The highest average classification accuracy obtained by each method on different feature sets was shown in bold

### Subtype classification

Table 2 showed the ACA and Var of 6 methods in 100 random data division experiments on the proposed feature set, $8^*$ feature set and whole feature set. $8^*$ feature set was first proposed in [16]. MIM, mRMR, CMIM, JMI, DISR, ICAP, CIFE, and CONDRED were used to obtain 8 subsets of miRNAs. Each subset was considered to make an ensemble by further categorizing the miRNAs as $1^*$–$8^*$. If a miRNA was present in all the 8 miRNA subsets, then it was categorized as $8^*$. The reliability of comparative classification accuracy was ensured under the same data division. MLR-R, MLR-L, and MLR were solved via the R package *glmnet*. SVM and NB were solved via the R package *e1071*. RF was solved via the R package *randomForest*. Compared with the $8^*$ feature set in [16] and whole feature set, 5 of the 6 methods achieved higher ACA on the proposed feature set, except RF. Although RF achieved the highest ACA of 76.80% on the $8^*$ feature set, 2.61% lower than MLR-R on the proposed feature set. We tested the hypothesis “there is no significant difference between the two results” with *t*-test. The obtained *p*-value was 1.711e$-$08, much less than 0.05. Therefore, the original hypothesis was rejected, i.e., the improvement in ACA from 76.8 to 79.41% was statistically significant. Furthermore, MLR-R achieved ACA of 77.15% higher than RF by 0.35% on the same $8^*$ feature set.

**Table 2:** ACA of 6 methods on different datasets

	124 miRNA$^{[proposed]}$	$8^*$ feature set [16]	The whole feature set
MLR-R	**0.7941** (0.0026)	0.7715 (0.0025)	0.7361 (0.0018)
MLR-L	**0.7274** (0.0030)	0.7185 (0.0027)	0.7167 (0.0023)
MLR	**0.6574** (0.0037)	0.6257 (0.0037)	0.6361 (0.0041)
RF	0.7504 (0.0020)	**0.7680** (0.0024)	0.7491 (0.0020)
SVM	**0.7657** (0.0021)	0.7554 (0.0019)	0.7415 (0.0024)
NB	**0.7565** (0.0028)	0.7285 (0.0033)	0.7198 (0.0039)

The highest average classification accuracy obtained by each method on different feature sets was shown in bold

### Identification of miRNA biomarkers

Cox regression based survival analysis was performed via R package *survival* on the proposed feature set. In the survival data, the censored data and the sample status still alive at the end of the follow-up time were recorded as 0, and the death status was recorded as 1. The 22 miRNAs corresponding to the conditions that the absolute value of the Cox regression coefficient was greater than 0.2 and the *p*-value was less than 0.05 were identified as breast cancer biomarkers. The identified miRNA biomarkers were listed in Table 3 according to the absolute value of the Cox regression coefficient. MiRNA corresponding to hazard ratio greater (less) than 1 will increase (reduce) the risk of death. For example, hsa-miR-130b-3p with high expression will increase the risk of death since its corresponding hazard ratio was 1.2479. On the contrary, hsa-miR-495-3p, hsa-miR-29a-3p, and hsa-miR-452-5p with high expression will reduce the risk of death due to the relatively small hazard ratio. The regulatory role of these miRNAs was also confirmed in the following section K–M survival analysis and expression analysis .

**Table 3:** The identified miRNA biomarkers

MiRNA	Cox coefficient	Hazard ratio	*p*-value
hsa-miR-30e-3p	−0.3138	0.7307	0.0381
hsa-miR-1266-5p	0.3046	1.3561	0.0007
hsa-miR-99b-5p	0.2803	1.3235	0.0083
hsa-miR-629-5p	0.2759	1.3177	0.0073
hsa-let-7e-5p	0.2713	1.3117	0.0233
hsa-miR-27b-5p	0.2526	1.2873	0.0177
hsa-let-7g-3p	0.2497	1.2836	0.0032
hsa-miR-125a-3p	0.2457	1.2785	0.0237
hsa-miR-193b-5p	0.2344	1.2642	0.0035
hsa-miR-99b-3p	0.2322	1.2614	0.0069
hsa-miR-744-5p	0.2248	1.2521	0.0202
hsa-miR-29a-3p	−0.2247	0.7988	0.0249
hsa-miR-107	0.2225	1.2492	0.0493
hsa-miR-130b-3p	0.2215	1.2479	0.0015
hsa-miR-495-3p	−0.2202	0.8024	0.0031
hsa-miR-331-3p	0.2149	1.2397	0.0157
hsa-miR-340-5p	0.2145	1.2392	0.0306
hsa-miR-127-3p	−0.2130	0.8082	0.0017
hsa-miR-671-5p	0.2089	1.2323	0.0114
hsa-miR-30a-5p	−0.2062	0.8137	0.0013
hsa-miR-452-5p	−0.2025	0.8167	3.09E−05
hsa-miR-889-3p	−0.2013	0.8177	0.0052

### Biological analysis

Kaplan–Meier (K–M) survival analysis [20], expression analysis, regulatory network analysis [21], Protein–Protein Interaction (PPI) analysis [22], Kyoto Encyclopedia of Genes and Genomes (KEGG) pathway analysis [23], Gene Ontology (GO) enrichment analysis [24], and miRNA–protein–drug interaction network were performed to analyze the rationality of the identified 22 miRNA. The detailed process and results were described in the following section.

#### K–M survival analysis and expression analysis

**Fig. 3:**
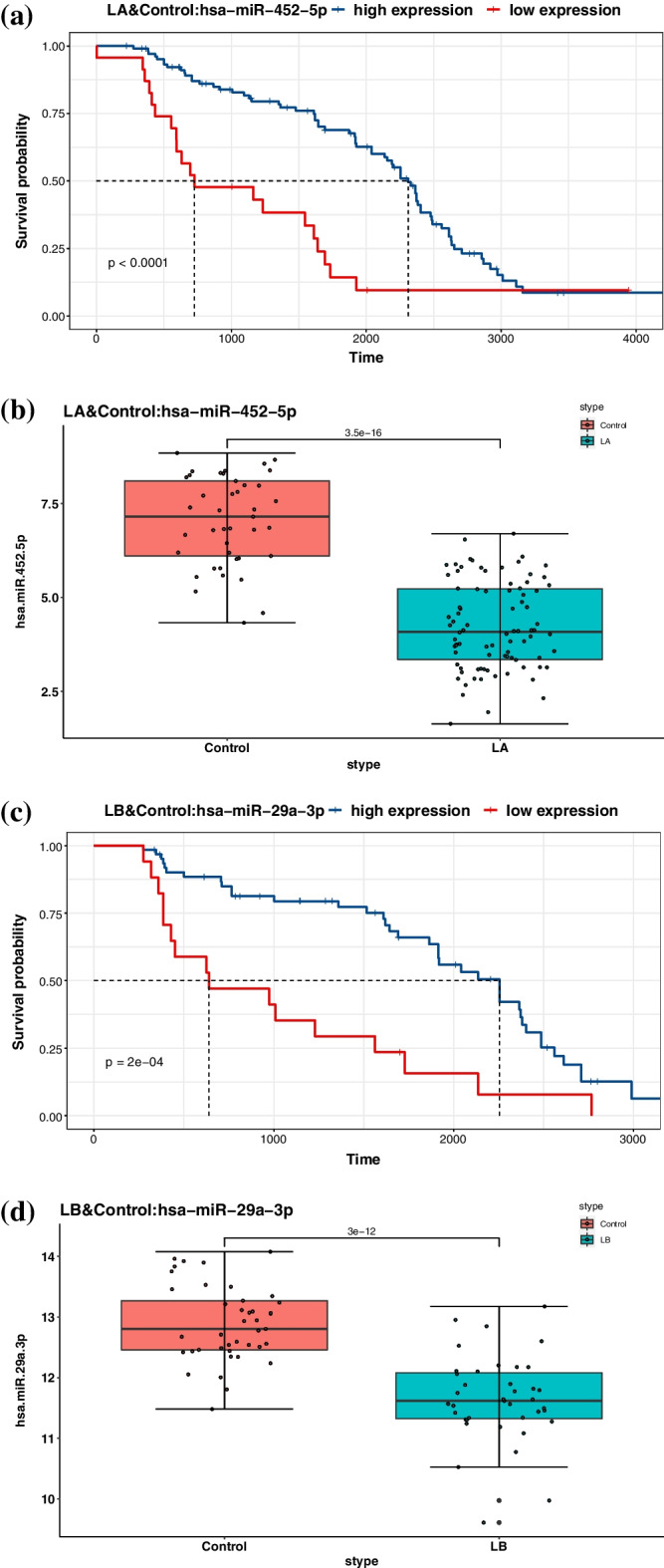

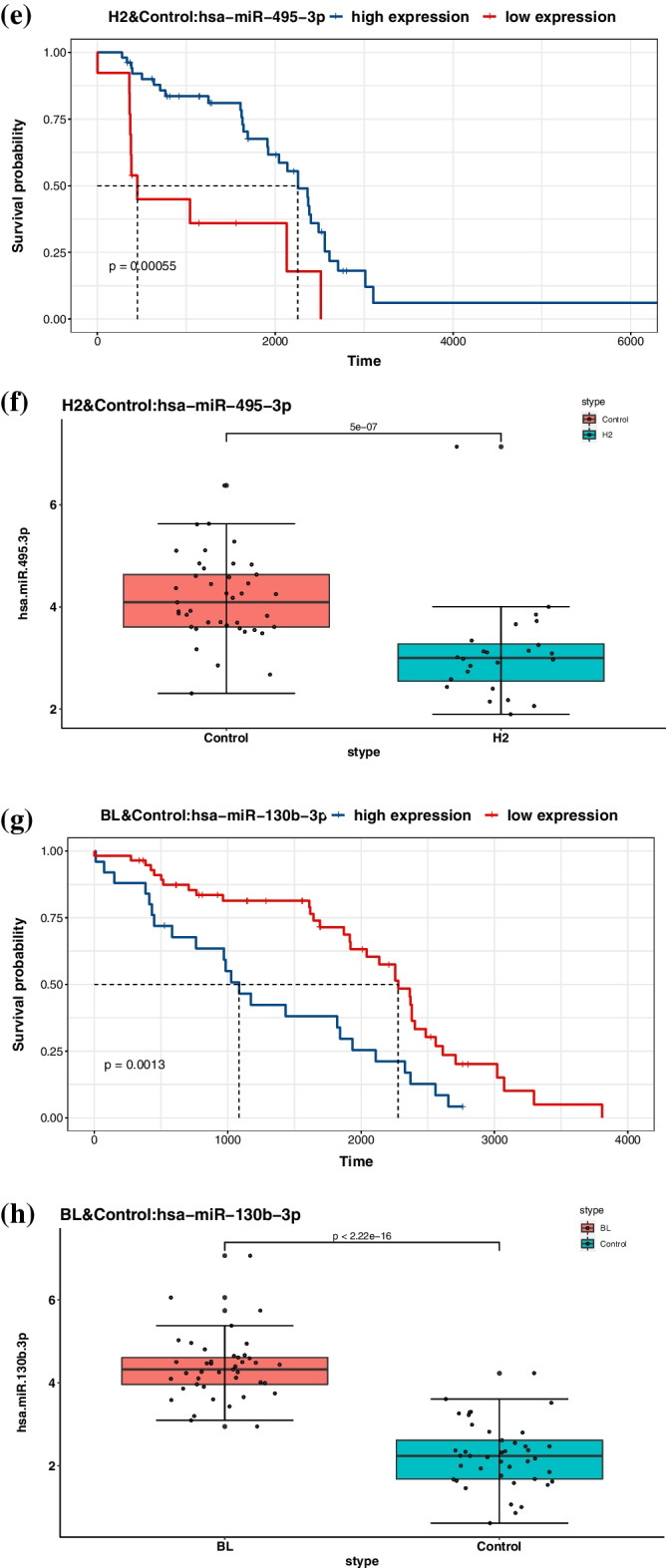
K–M survival analysis and expression analysis

We screened the subset of the identified miRNAs that simultaneously participate in the control subtype and another subtype and performed K–M survival analysis and expression analysis. K–M survival analysis was implemented via R package *survival* and *survminer*, and the log-rank test was performed to obtain *p*-values. For LA, LB, H2, and BL, only one of the analysis results of each subtype were listed in Fig. 3. The remaining 34 analysis results were shown in Additional file 1: Figs. S1–S6.

Figure 3 showed that the identified miRNA could significantly distinguish the survival probability of the low and high expression group, which can be used as a factor for prognosis inference. In addition, the expression value of miRNA was shown through the box plot, and the *p*-value reflected the significant difference between the control subtype and another subtype. These analyses confirmed the facts published in the references. For example, hsa-miR-130b-3p was found to have crucial relevance for breast cancer biology, and its expression was up-regulated [25]. Hsa-miR-452-5p had a tumor suppressive role, and its declining expression level will promote the metastasis of breast cancer [26]. Moreover, the low expression of miR-29a-3p was associated with lower overall breast cancer survival [27]. Hsa-miR-495-3p was also down-regulated in the early stages of breast cancer [28].

#### Regulatory network analysis

**Fig. 4:**
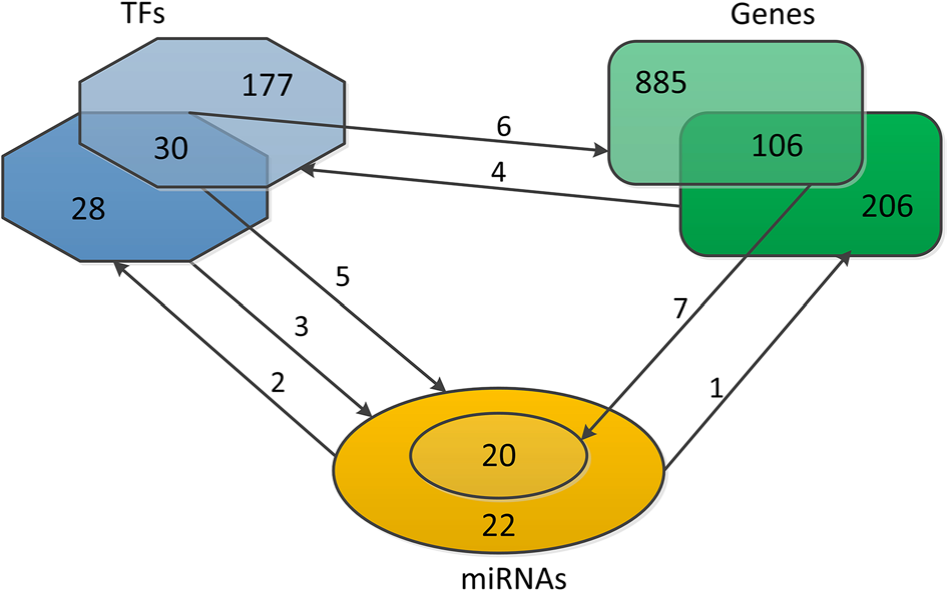
Illustration of the steps of refining the miRNAs, gene and TF sets

Transcription factors (TFs) and target genes related to 22 miRNA were found through miRTarbase [29], TRRUST [30], and TransmiR [31] databases. Fig 4 visualized the following detailed steps.

1. Identify 312 target genes associated with identified 22 miRNAs.
2. Identify 58 TFs that target 22 miRNAs.
3. Verify that all 22 miRNAs were targeted by 58 TFs.
4. Identify 207 key TFs through 312 genes obtained from step 1.
5. Verify that all 22 miRNAs were targeted by 30 TFs obtained from steps 2 and 4.
6. Identify 991 genes targeted by 30 TFs obtained from steps 2 and 4.
7. Identify 20 miRNAs that target 106 genes obtained from steps 1 and 6.

**Fig. 5:**
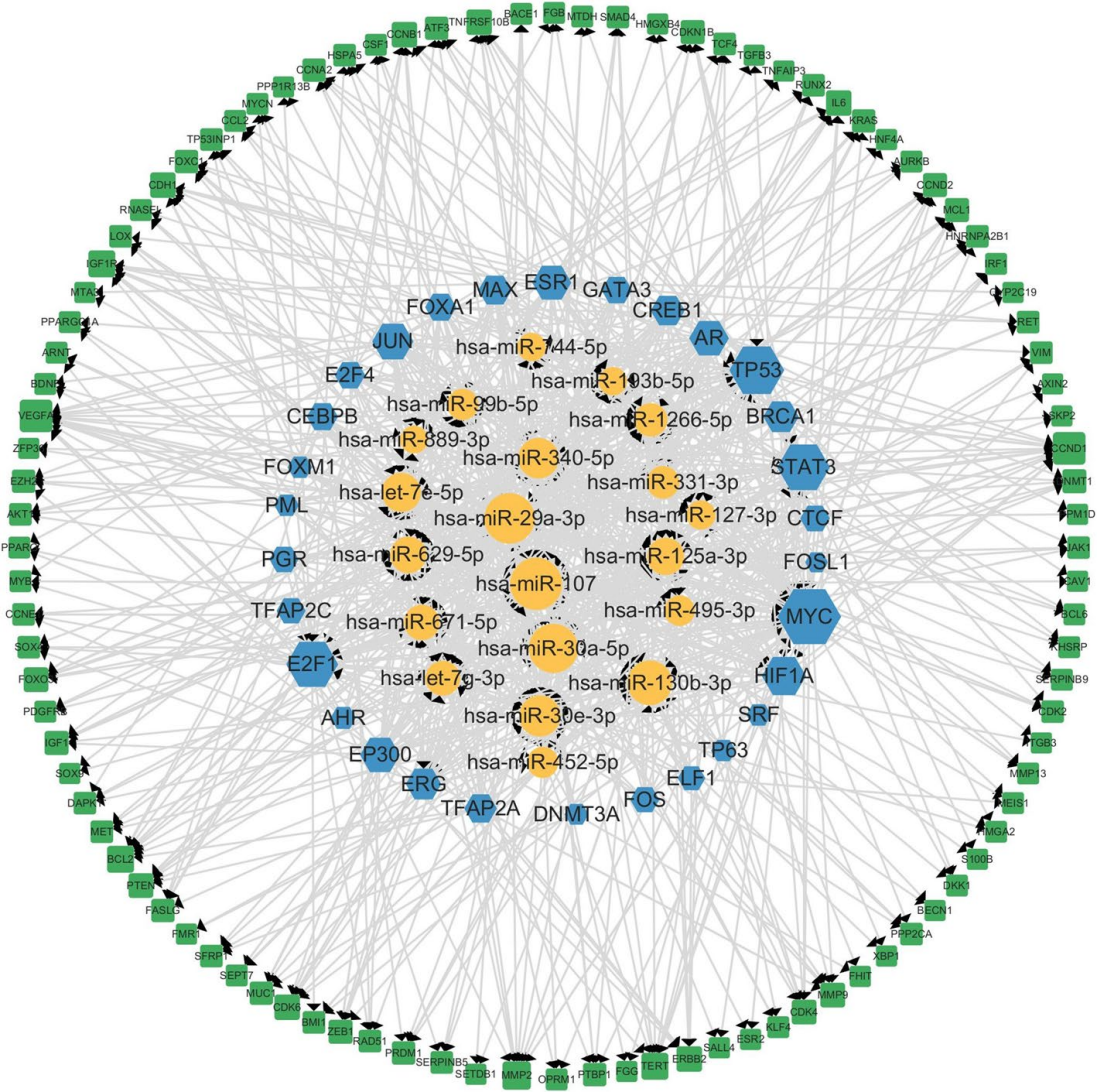
Global regulatory networks of miRNAs (yellow), genes (green) and TFs (blue)

**Fig. 6:**
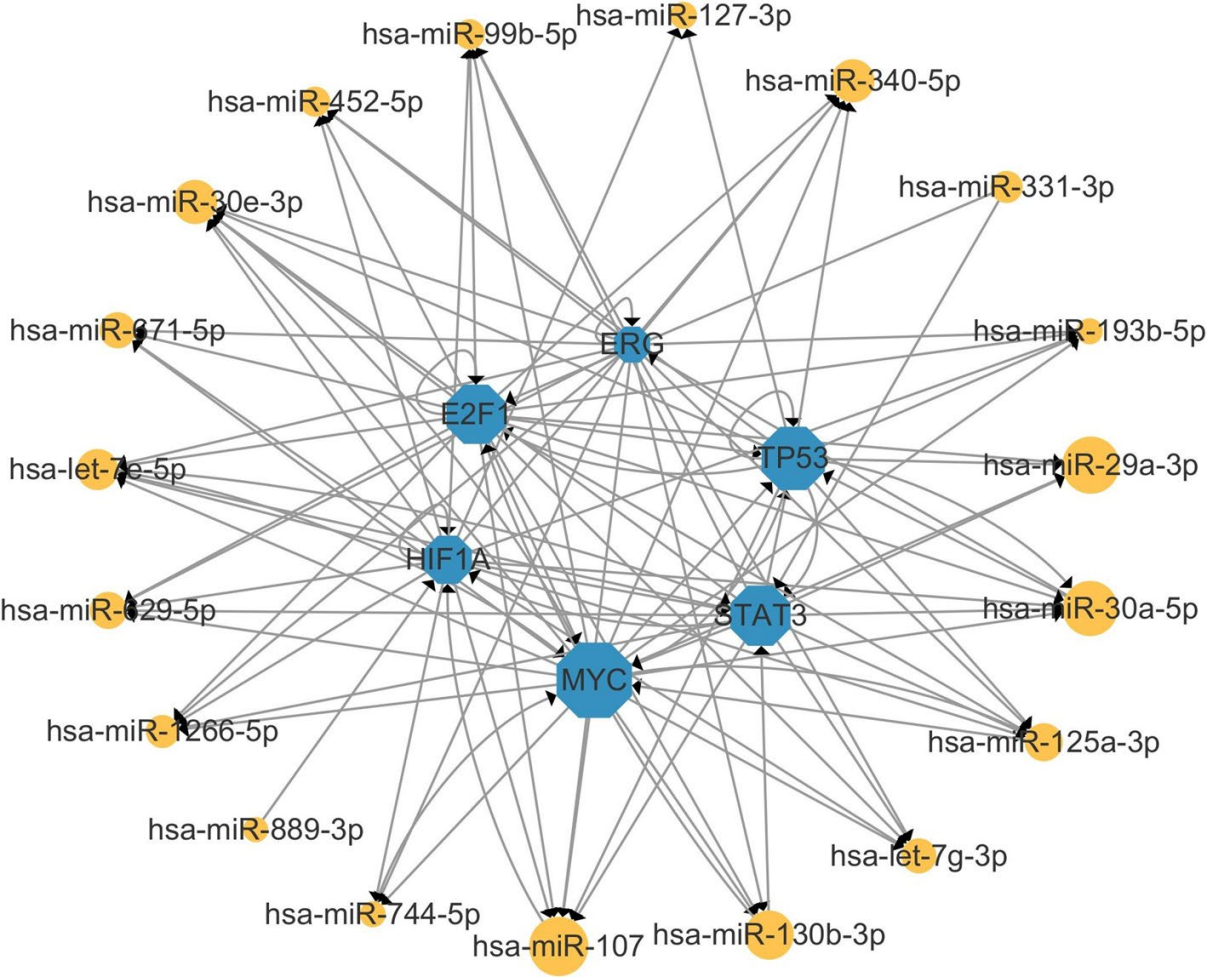
Subnetwork of containing the loops between miRNAs and same-named genes and TFs

Subsequently, the regulatory networks of miRNA, TFs, and genes were constructed via the Cytoscape tool. Figure 5 showed the global regulatory network. The size of the node area in the Fig. 5 represents the degree of the node. For larger nodes, hsa-miR-107, hsa-miR-30a-5p, hsa-miR-29a-3p, and hsa-miR-130b-3p have been proved to suppress or drive the occurrence of breast cancer [25, 27, 32, 33]. In order to more clearly show the regulatory relationship between miRNAs, TFs, and genes, those genes and TFs with the same name were selected to construct subnetworks. It was observed in Fig 6 that MYC and TP53 had more connections with miRNA and were considered oncogenes for breast cancer with poor prognosis [34, 35].

#### PPI network analysis

**Fig. 7:**
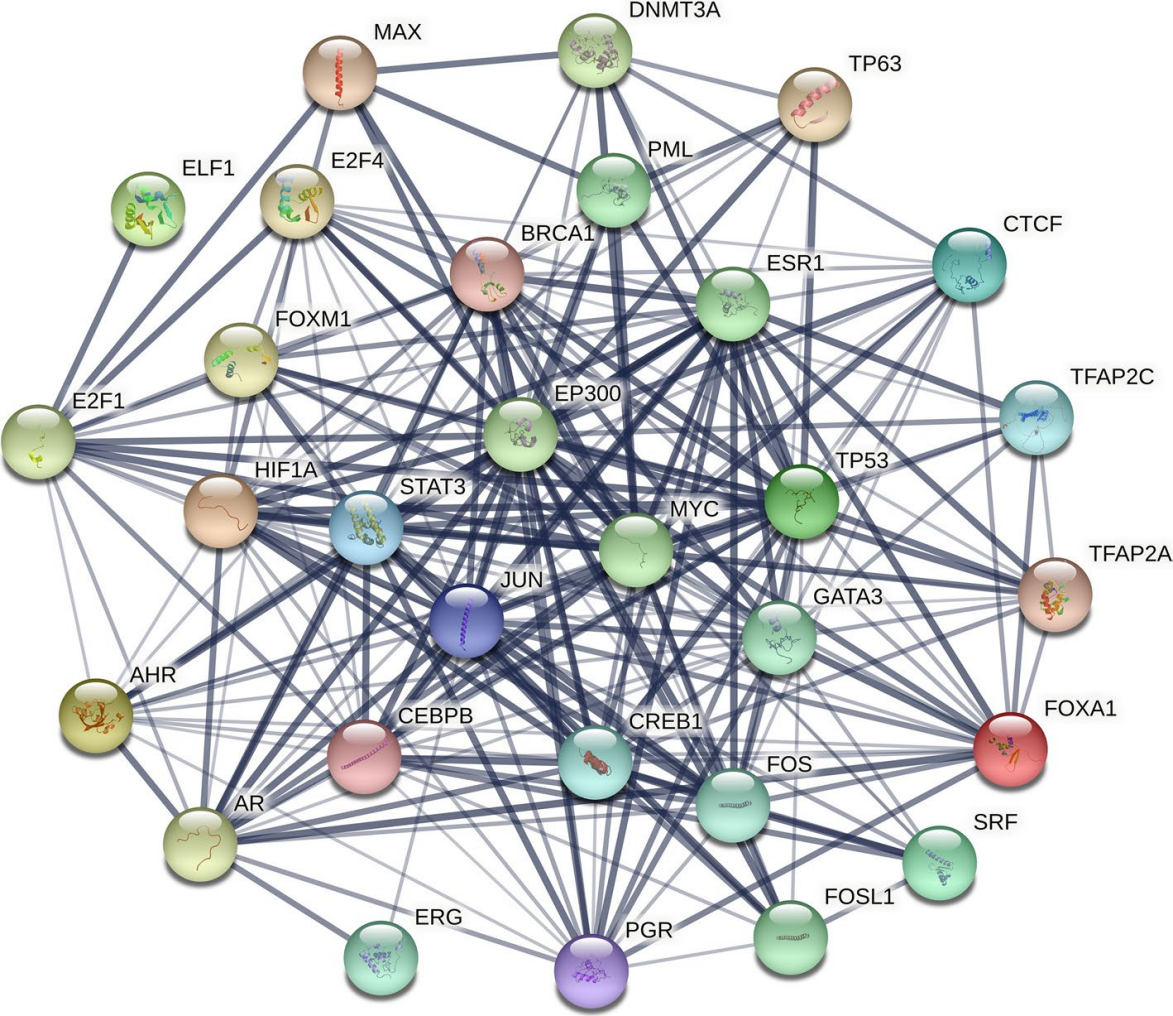
PPI network analysis

Proteins cannot independently perform their unique biological significance, and they rely on interactions to achieve their significant impact [36]. PPI networks of 30 TFs targeting 22 miRNA were constructed through STRING database [22]. Details of the interaction between TFs were shown in Fig. 7. Each edge of the network indicates both functional and physical protein associations, and the line thickness indicates the strength of data support. In addition, the *p*-value of this PPI network was less than 1.0E−16 and the average degree node was 15.2. The TFs in the top 5 degrees were listed in Table 4. EP300 was a tumor suppressor, down-regulated in metaplastic breast cancer [37]. Moreover, BRCA1 was considered a tumor suppressor gene. When BRCA1 mutates, it is associated with the occurrence of hereditary breast cancer [38]. Similarly, the expression of MYC, TP53, and JUN was also closely related to breast cancer [34, 35, 39]. These evidences illustrated that the identified 22 miRNAs belong to the breast cancer pathway.

**Table 4:** TFs in the top 5 degrees of nodes

Node	EP300	MYC	TP53	JUN	BRCA1
Node-degree	27	27	25	24	23

#### KEGG pathway analysis

The 106 genes targeted by 20 miRNAs were subjected to KEGG pathway analysis through ConsensusPathDB [40] database. The parts of pathway name in which genes participate and the *p*-value were listed in Table 5. The pathways in Table 5 have been annotated by many documents [41–43]. PI3K-Akt signaling pathway played an essential role in the pathogenesis of breast cancer, regulating cell proliferation, metabolism, and other vital functions [41]. FoxO signaling pathway can be used as a cancer treatment target to find and develop some effective drugs for cancer [42]. Molecular pathological analysis of the p53 signaling pathway was considered valuable in the diagnosis, prognosis evaluation, and final treatment of breast cancer [43]. In addition, it was observed from the Table 5 that Pathways in cancer, MicroRNAs in cancer, and Breast cancer were significantly correlated with breast cancer. These results demonstrated that the identified miRNA sets could be potential biomarkers.

**Table 5:** KEGG pathway analysis

Pathway	*p*-value
Pathways in cancer	1.7E$-$29
PI3K-Akt signaling pathway	3.7E$-$22
MicroRNAs in cancer	2.7E$-$21
FoxO signaling pathway	6.3E$-$18
p53 signaling pathway	2.9E$-$16
Breast cancer	6.4E$-$12

#### GO enrichment analysis

**Fig. 8:**
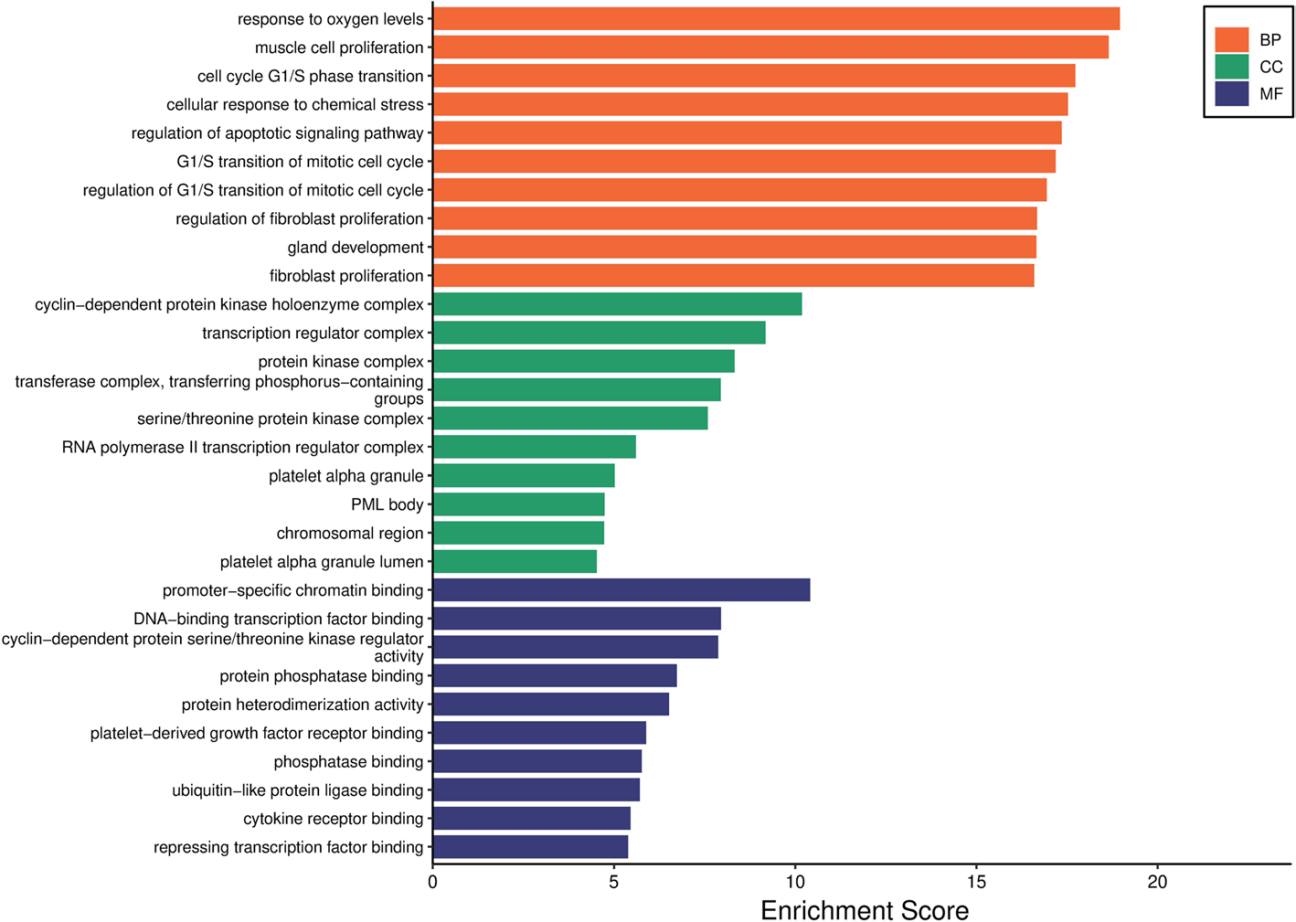
GO results of three ontologies

Further, GO enrichment analysis was performed by bioinformatics online tool (http://www.bioinformatics.com.cn/?p=1) for the 106 genes. GO includes 3 ontologies, biological process (BP), cellular component (CC), and molecular function (MF), which can comprehensively describe the attributes of genes and gene products in organisms [24]. Figure 8 showed that the 10 terms in each type of ontology. The horizontal axis represents the enrichment fraction taken as $-log10$(*p*-value), and the vertical axis represents the functional description of the GO term. It was observed from the figure that some significant GO terms related to BP mainly enriched in response to oxygen levels (GO:0070482, *p* = 1.11E$-$19), muscle cell proliferation (GO:0033002, *p* = 2.27E$-$19), and cell cycle G1/S phase transition (GO:0044843, *p* = 1.88E$-$18). Meanwhile, cyclin-dependent protein kinase holoenzyme complex (GO:0000307, *p* = 6.53E$-$11), transcription regulator complex (GO:0005667, *p* = 6.59E$-$10), and protein kinase complex (GO:1902911, *p* = 4.70E$-$09) were significantly enriched in CC. Similarly, among MF, the important enriched GO terms were promoter-specific chromatin binding (GO:1990841, *p* = 3.91E$-$11), DNA-binding transcription factor binding (GO:0140297, *p* = 1.12E$-$08), and cyclin-dependent protein serine/threonine kinase regulator activity (GO:0016538, *p* = 1.34E$-$08).

#### MiRNA–protein–drug interaction network analysis

**Fig. 9:**
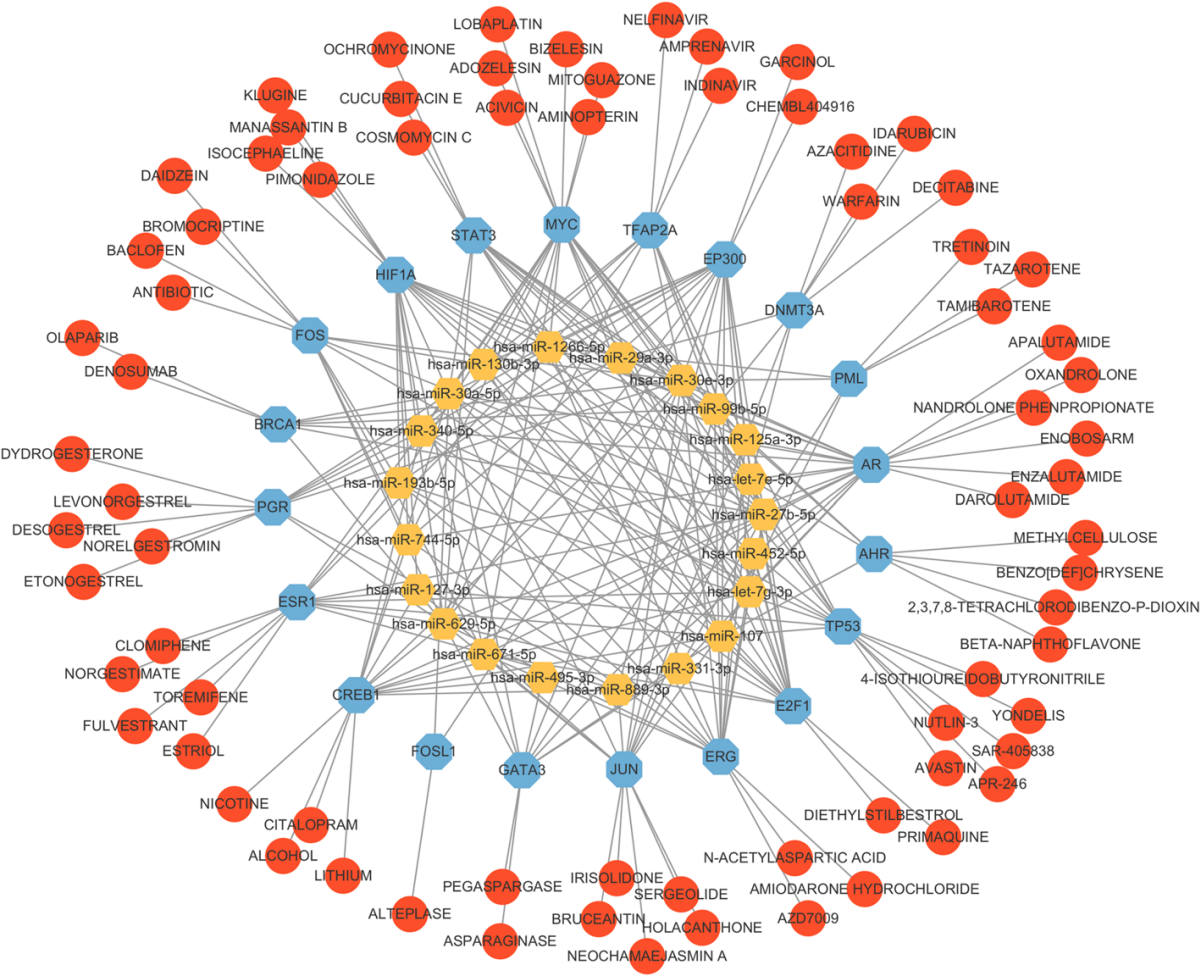
MiRNA–protein–drug interaction network

MiRNAs are closely associated with diseases [44]. In order to make the identified 22 miRNAs more clinically significant, we studied potential drugs that regulate them. However, treating diseases based on drugs directly targeting miRNA still faces many challenges and may lead to unpreventable consequences [45, 46]. Fortunately, drugs targeting proteins involved in this miRNA pathway can regulate the function of miRNA [45]. Motivated by this idea, we constructed the miRNA–protein–drug interaction network through BioGRID database [47]. Figure 9 showed that the details of the interaction between miRNA (yellow), protein (blue), and drug (red).

Drug samples {GARCINOL}, {LOBAPLATIN, AMINOPTERIN, ACIVICIN, MITOGUAZONE, ADOZELESIN}, {APR-246, NUTLIN-3, AVASTIN, YONDELIS}, {SERGEOLIDE, BRUCEANTIN, HOLACANTHONE}, and {OLAPARIB, DENOSUMAB} target significant proteins EP300, MYC, TP53, JUN, and BRCA1. It has been found to be related to the treatment of breast cancer [48–53]. For example, GARCINOL achieves anticancer activity against breast cancer cells by regulating epithelial-to-mesenchymal transition and Wnt signaling pathways [48]. Oral OLAPARIB has shown clinical efficacy in phase III clinical trials for treating mutant BRCA-positive HER2 negative metastatic breast cancer [51]. In addition, although there is no direct evidence that SAR-405838 and BIZELESIN can be used in the treatment of breast cancer, they can be used in the phase I study of patients with advanced malignant tumors [54, 55].

## Discussion

For the identified 22 miRNAs, we performed a bibliographic meta-analysis of specialized literature. Among them, hsa-miR-495-3p [28], hsa-miR-30a-5p [33], hsa-let-7e-5p [56], hsa-let-7g-3p [57], hsa-miR-127-3p [58], hsa-miR-30e-3p [59], hsa-miR-340-5p [60], hsa-miR-193b-5p [61], and hsa-miR-744-5p [62] have been considered biomarkers for breast cancer by previous literature. In addition, LINC0092 was considered a therapeutic marker for breast cancer and was regulated by hsa-miR-452-5p [26]. Hsa-miR-130b-3p [25], hsa-miR-29a-3p [27], hsa-miR-107 [32], hsa-miR-99b-5p [63], hsa-miR-99b-3p [64], hsa-miR-27b-5p [65], hsa-miR-125a-3p [66], hsa-miR-331-3p [67], hsa-miR-629-5p [68], hsa-miR-671-5p [69], hsa-miR-889-3p [70], and hsa-miR-1266-5p [71] have been proved to be highly related to breast cancer. These 12 miRNAs may be considered potential biomarkers for breast cancer, providing a new idea for related research.

We selected the most suitable sample subset to reduce data division’s impact on miRNA feature screening. In addition, we also performed Algorithm 1 on all sample sets and obtained 122 miRNA as a feature set. MLR-R achieved ACA of 79.93% on this feature set, which was 0.52% higher than that on the proposed feature set. From the perspective of subtype classification, feature screening should be based on the training dataset, and a test set should be reserved in advance to verify the prediction ability. This is why we do not use all sample information to screen features. After considering the classification accuracy, algorithm execution time, and sample size, we set the number of integrations of learners (MLR-EN) to 100. As for the comparison of classification results of 6 methods on 3 different feature sets, we set 1–100 continuous random seeds to ensure that each method was tested under the same data division. Although 10-fold cross-validation can accurately obtain the error estimation of the model to a certain extent, the results change with the change of random seeds. The proposed ACA of 6 methods in 100 experiments showed the actual results more fairly.

We considered the normal samples as the control subtype, i.e., the fifth subtype. According to the non-zero regression coefficients that appeared more than 50 times in 100 MLR-EN models, 128 miRNAs that participated in control subtype classification were identified. Among the 128 miRNAs, 8 miRNAs only participated in the classification of control subtypes. Therefore, genes regulated by 8 miRNAs were thought to participate in the classification of the control group. These genes can be identified through miRTarbase database. As this is not the paper’s focus, we do not present detailed results.

We combined the survival information of the sample to perform Cox regression analysis on the proposed feature set. From the perspective of statistics, the miRNAs corresponding to $p<0.05$ were significantly correlated with survival. Moreover, we set the threshold of the absolute value of the regression coefficient to be 0.2. The choice of thresholds was derived from the analysis of experimental results. 18 miRNAs were selected if the threshold was 0.21. Compared with the results of the proposed threshold, hsa-miR-30a-5p, hsa-miR-452-5p, hsa-miR-671-5p, and hsa-miR-889-3p were excluded. However, hsa-miR-889-3p has been proved to be a biomarker for breast cancer. In particular, LINC0092 was considered a therapeutic marker for breast cancer and was regulated by hsa-miR-452-5p. This indicates that hsa-miR-452-5p has a great possibility of being a breast cancer biomarker. Conversely, 7 miRNAs would be increased if the threshold was set to 0.19. Through a literature search, there was no evidence showing that these 7 miRNAs were closely associated with breast cancer. Combined with the condition $p<0.05$ and threshold limit, 22 miRNA were identified. For these 22 miRNAs, we analyzed the miRNA, the targeted genes, the TFs, and the related drugs to determine the rationality of the identified 22 miRNAs as potential biomarkers of breast cancer from multiple perspectives.

The excellent performance of this article could be attributed to five factors:

- Based on stratified sampling and bootstrap sampling, the most suitable sample subset for miRNA feature screening was determined via ensemble MLR-EN.
- In the screening process of 124 features, both the number of subtypes that miRNA participates in classification and the frequency of miRNA that appeared in 100 integrations were fully considered.
- 22 miRNAs from the proposed feature set were further identified as breast cancer biomarkers by using Cox regression based on survival analysis.
- The identified 22 miRNAs were analyzed from multiple perspectives to the rationality.
- The subtype classification performance of the 6 methods was measured fairly and objectively through the proposed comparison method.

Undeniably, the coefficient threshold of Cox regression are empirical values in our method, and different data may have different suitable values. Moreover, our proposed method is based on ensemble learning, whose execution time will be significantly longer than traditional machine learning methods. In addition, we only use one dataset without looking for external datasets for verification. In future work, we will collect more data to verify our results.

## Conclusions

In this paper, we combined ensemble regularized multinomial logistic regression and Cox regression to identify miRNA biomarkers in breast cancer. 124 miRNAs meeting specific conditions were screened as feature set. 6 methods on 3 different feature sets were fairly compared, and the proposed features can significantly improve the subtype classification accuracy of 5 methods. Based on this feature set, 22 miRNA biomarkers were identified by performing Cox regression analysis. Subsequently, K–M survival analysis, expression analysis, regulatory network analysis, PPI analysis, KEGG pathway analysis, GO enrichment analysis, and miRNA–protein–drug interaction network were performed to analyze the rationality of the identified biomarkers from multiple perspectives.

In particular, possible drugs were suggested through miRNA–protein–drug interaction network. Of course, these drugs must be further studied for solid clinical evidence. All relevant drugs were not fully shown in Fig. 9 to make the results more observable. In this event, 15 drug samples targeting 5 significant proteins have been found to have the function of treating breast cancer. This evidence provides more support for 22 miRNAs as potential biomarkers of breast cancer. In a word, the results of this paper included well-known and underestimated miRNAs, which may provide clues for some related studies.

## Methods

### Data preparation

The high throughput next-generation sequencing data of miRNA expression of Breast Invasive Carcinoma were downloaded from The Cancer Genome Atlas [72]. This dataset includes 231 samples and 587 miRNAs, including 86 LA, 39 LB, 24 H2, 41 BL, and 41 control subtypes. Accordingly, each sample has clinical information such as survival time and status. However, many miRNAs in the data have no expression values in some samples. Sarkar et al. cleared miRNA with the non-expression value of more than 1% and normalized the remaining miRNA expression value by taking the logarithm of base 2. Based on this, 296 miRNAs were obtained [16]. In order to make the dataset look clearer, we listed the information of 5 subtypes in Table 6.

**Table 6:** The information of 5 subtypes

Sample	Sample size	No. of miRNA
LA	86	296
LB	39	296
H2	24	296
BL	41	296
Control	41	296

### Method description

Given miRNA expression profiling data $\{(x_1,y_1),\ldots ,(x_i,y_i),\ldots ,(x_{231},y_{231})\}$, where $x_i=(x_{i1},x_{i2},\ldots ,x_{i296})^T$ denotes the expression levels of 296 miRNAs for the *i*th sample, $y_i$ represents a subtype label corresponding to $x_i$. If the *i*th sample comes from LA, LB, H2, BL, or control, $y_i$ takes 1, 2, 3, 4, or 5 accordingly. We randomly selected 4/5 (185) of the samples to train the model, the rest to test the model. Stratified sampling was performed to avoid a small number of subtypes not being selected for the training set.

#### Multinomial logistic regression

Logistic regression is a classical binary machine learning method that has achieved many results in cancer diagnosis [73, 74]. When the sample category exceeds two classes, the logistic regression can be generalized to the multinomial logistic regression [75]. Distinguishing breast cancer subtypes is considered a 5 classification task in this paper. The maximum log-likelihood of multinomial logistic regression is written as the following function:

1 $$\begin{aligned} \begin{aligned} l(w,b)=\sum _{i=1}^{231}\left[ \sum _{j=1}^5\mathbf {I}(y_i=j)(w^Tx_i+b)-\log \left( \sum _{j=1}^5e^{w^Tx_i+b}\right) \right] , \end{aligned} \end{aligned}$$

where $\mathbf {I}(\cdot )$ is the indicator function, $y_i\in \{1,2,3,4,5\}$ denotes subtype label, $w=(w_1,w_2,\ldots ,w_{296})^T$ is the regression coefficient vector and *b* is the offset.

#### Model building

By combining multinomial log-likelihood loss and elastic net penalty, we proposed the following multinomial logistic regression with elastic net penalty model:

2 $$\begin{aligned} \begin{aligned} <\bar{w},\bar{b}>=\arg \min _{w,b}\left\{ -\frac{1}{231}l(w,b)+\lambda P_{\alpha }(w)\right\} , \end{aligned} \end{aligned}$$

where $\lambda$ is the regularization parameter, $P_{\alpha }(w)=(1-\alpha )\frac{1}{2}\sum _{k=1}^{296}w_k^2+\alpha \sum _{k=1}^{296}|w_k|$ is elastic net penalty, $\alpha \in [0,1]$ denotes the regularization parameter, and $w_k$ represents the regression coefficient corresponding to the *k*th miRNA. Elastic net penalty is a popular feature selection method in bioinformatics [76, 77]. When $\alpha =0$, (2) is MLR-R. When $0<\alpha <1$, (2) is MLR-EN. When $\alpha =1$, (2) is MLR-L.

Bootstrap sampling was performed 100 times on the training set, and learners (MLR-EN) were ensembled. MiRNAs that appeared at least 50 times in 100 integrations were screened to ensure the rationale of the voting strategy. The selected miRNA subset corresponding to the *j*th subtype is defined as follows:

3 $$\begin{aligned} D_j= & {} \left\{ q_k|\sum _{m=1}^{100}\mathbf {I}(\bar{w}_{k,j}^m\ne 0)\ge 50\right\} , \end{aligned}$$

4 $$\begin{aligned} D= & {} \{D_1,D_2,D_3,D_4,D_5\}, \end{aligned}$$

where $q_k$ represents the *k*th miRNA, $\bar{w}_{k,j}^m$ represents the regression coefficient corresponding to the *k*th miRNA in the *j*th subtype obtained by the *m*th learner, and *D* contains the results of 5 subtypes.

In addition, we consider that miRNA participation in at least half of the subtypes is more important. Therefore, we further process the miRNA subset obtained by (3) and select features that participated in at least 3 subtypes:

5 $$\begin{aligned} \begin{aligned} D^*=\left\{ q_k|\sum _{j=1}^{5}\mathbf {I}(\bar{w}_{k,j}\ne 0)\ge 3\right\} , \end{aligned} \end{aligned}$$

where $\bar{w}_{k,j}$ represents the regression coefficient corresponding to the *k*th miRNA participating in the *j*th subtype in *D*, $q_k$ is the same as described in (3).

#### Cox regression

Cox proportional hazard model can study the relationship between risk factors and patient survival. The formula of Cox regression is as follows:

6 $$\begin{aligned} \begin{aligned} \frac{h(t)}{h_0(t)}=e^{\beta _1x_1+\cdots +\beta _{124}~~x_{124}}, \end{aligned} \end{aligned}$$

where *h*(*t*) is the risk function, $h_0(t)$ is the baseline risk function, $\frac{h(t)}{h_0(t)}$ represents the hazard ratio, and $(\beta _1,\ldots ,\beta _{124})^T$ is the regression coefficient vector corresponding to 124 miRNAs in $D^*$.

#### Algorithm

The steps of determining the most suitable sample subset for miRNA feature screening were shown in Algorithm 1. Moreover, the algorithm steps of solving ensemble regularized multinomial logistic regression were shown in Algorithm 2:
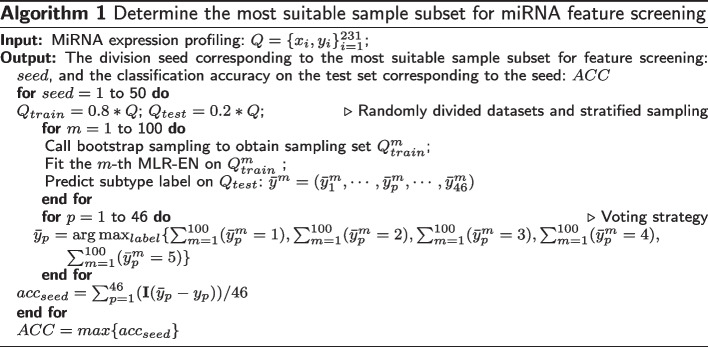

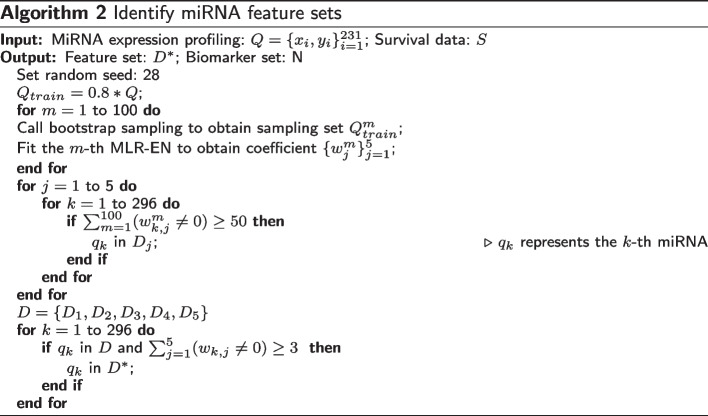


## Supplementary Information

**Additional file 1: Table S1**. Prediction accuracy and cross entropy loss results of 50 data division experiments. **Table S2**. Names of 124 miRNAs participated in classification of at least 3 subtypes. **Figures S1–S6**. K–M survival analysis and expression analysis of miRNAs that simultaneously participate in the control subtype and another subtype.

## Abbreviations

LA: Luminal A
LB: Luminal B
H2: HER2-Enriched
BL: Basal-Like
RF: Random forest
miRNA: microRNA
MLR-EN: Multinomial logistic regression with elastic net penalty
MLR: Multinomial logistic regression
MLR-R: Multinomial logistic regression with ridge regression penalty
MLR-L: Multinomial logistic regression with lasso penalty
SVM: Support vector machine
NB: Naive Bayes
K–M: Kaplan–Meier
PPI: Protein–Protein Interaction
KEGG: Kyoto Encyclopedia of Genes and Genomes
GO: Gene Ontology
ACA: Average classification accuracy
Var: Variance
TFs: Transcription factors
BP: Biological process
CC: Cellular component
MF: Molecular function

## Declarations

### Availability of data and materials

The datasets used and analyzed during the current study are available in the Cancer Genome Atlas (TCGA, https://cancergenome.nih.gov/) database. The dataset supporting the conclusions of this article is included within the article and its additional file. The codes used are available from the corresponding author on reasonable request.
