# Supplementary material for: Identification of miRNA biomarkers for breast cancer by combining ensemble regularized multinomial logistic regression and Cox regression
Source: BMC Bioinformatics. 2022 Oct 18;23:434. doi: 10.1186/s12859-022-04982-7 (PMC9580207; doi:10.1186/s12859-022-04982-7)
Supplement: Supplementary file 1 — Additional file 1: Table S1. Prediction accuracy and cross entropy loss results of 50 data division experiments. Table S2. Names of 124 miRNAs participated in classification of at least 3 subtypes. Figures S1–S6. K–M survival analysis and expression analysis of miRNAs that simultaneously participate in the control subtype and another subtype. [file 12859_2022_4982_MOESM1_ESM.pdf]

SUPPLEMENTARY INFORMATION

Identification of miRNA biomarkers for breast cancer by combining ensemble regularized multinomial logistic regression and Cox regression

Juntao Li, Hongmei Zhang* and Fugen Gao

*Correspondence:
zhanghmmail@163.com
College of Mathematics and Information Science, Henan Normal University, Xinxiang, China
Full list of author information is available at the end of the article

1 Supplementary Tables

Supplementary Table 1 Prediction accuracy and cross entropy loss results of 50 data division experiments

Seed	Accuracy	Cross loss entropy	Seed	Accuracy	Cross loss entropy
1	0.8043	0.8903	26	0.7391	1.0574
2	0.7391	1.0833	27	0.8043	0.7135
3	0.7609	1.4357	**28**	**0.9130**	**0.4777**
4	0.7391	0.9580	29	0.7609	0.7332
5	0.8043	0.8113	30	0.8478	0.5321
6	0.6957	1.2737	31	0.8043	0.7316
7	0.7391	1.3124	32	0.8478	0.6789
8	0.6739	1.3070	33	0.7609	0.8076
9	0.7391	1.0713	34	0.7391	0.8883
10	0.7826	0.7552	35	0.7826	1.2385
11	0.7609	1.1144	36	0.8261	0.6470
12	0.8261	0.7677	37	0.7826	1.0695
13	0.8043	0.7440	38	0.7826	0.9786
14	0.7826	0.6022	39	0.6304	1.2511
15	0.7391	0.9656	40	0.6957	1.2875
16	0.7609	0.7904	41	0.7826	0.8485
17	0.8043	0.8170	42	0.6957	0.9732
18	0.7826	0.7750	43	0.7174	0.7618
19	0.7174	1.2278	44	0.7609	1.3971
20	0.8261	0.5780	45	0.7609	0.9417
21	0.6957	1.1401	46	0.7826	0.9391
22	0.7391	1.0506	47	0.7174	1.2761
23	0.7391	1.0924	48	0.7609	0.8259
24	0.8261	0.8949	49	0.7174	1.1528
25	0.7826	0.8749	50	0.7609	0.7266

Supplementary Table 2 Names of 124 miRNAs participated in classification of at least 3 subtypes

Name	Name	Name	Name
hsa-let-7a-5p	hsa-miR-10a-5p	hsa-miR-200a-3p	hsa-miR-181d-5p
hsa-let-7b-5p	hsa-miR-181c-5p	hsa-miR-301a-3p	hsa-miR-501-3p
hsa-let-7e-5p	hsa-miR-181c-3p	hsa-miR-99b-5p	hsa-miR-503-5p
hsa-miR-17-5p	hsa-miR-182-5p	hsa-miR-99b-3p	hsa-miR-505-3p
hsa-miR-17-3p	hsa-miR-183-5p	hsa-miR-130b-3p	hsa-miR-455-5p
hsa-miR-19b-1-5p	hsa-miR-203a-3p	hsa-miR-30e-3p	hsa-miR-539-5p
hsa-miR-20a-5p	hsa-miR-210-3p	hsa-miR-362-5p	hsa-miR-574-3p
hsa-miR-21-5p	hsa-miR-217	hsa-miR-374a-3p	hsa-miR-582-5p
hsa-miR-21-3p	hsa-miR-222-5p	hsa-miR-375	hsa-miR-582-3p
hsa-miR-22-5p	hsa-miR-224-5p	hsa-miR-340-5p	hsa-miR-584-5p
hsa-miR-24-2-5p	hsa-miR-200b-5p	hsa-miR-330-3p	hsa-miR-598-3p
hsa-miR-25-3p	hsa-miR-200b-3p	hsa-miR-328-3p	hsa-miR-625-3p
hsa-miR-26a-5p	hsa-let-7g-3p	hsa-miR-342-5p	hsa-miR-629-5p
hsa-miR-27a-3p	hsa-miR-1-3p	hsa-miR-342-3p	hsa-miR-653-5p
hsa-miR-29a-5p	hsa-miR-27b-5p	hsa-miR-337-3p	hsa-miR-542-3p
hsa-miR-29a-3p	hsa-miR-27b-3p	hsa-miR-326	hsa-miR-671-5p
hsa-miR-30a-5p	hsa-miR-30b-5p	hsa-miR-331-3p	hsa-miR-671-3p
hsa-miR-30a-3p	hsa-miR-132-3p	hsa-miR-338-3p	hsa-miR-320b
hsa-miR-32-5p	hsa-miR-143-5p	hsa-miR-339-3p	hsa-miR-454-3p
hsa-miR-93-5p	hsa-miR-144-5p	hsa-miR-423-3p	hsa-miR-889-3p
hsa-miR-93-3p	hsa-miR-191-5p	hsa-miR-425-5p	hsa-miR-708-3p
hsa-miR-96-5p	hsa-miR-191-3p	hsa-miR-425-3p	hsa-miR-744-5p
hsa-miR-101-5p	hsa-miR-125a-3p	hsa-miR-429	hsa-miR-1180-3p
hsa-miR-29b-2-5p	hsa-miR-126-5p	hsa-miR-451a	hsa-miR-1266-5p
hsa-miR-106a-5p	hsa-miR-127-3p	hsa-miR-452-5p	hsa-miR-1307-3p
hsa-miR-107	hsa-miR-134-5p	hsa-miR-486-5p	hsa-miR-3065-3p
hsa-miR-196a-5p	hsa-miR-149-5p	hsa-miR-511-5p	hsa-miR-378c
hsa-miR-148a-5p	hsa-miR-193a-5p	hsa-miR-493-5p	hsa-miR-3613-5p
hsa-miR-30c-5p	hsa-miR-106b-5p	hsa-miR-432-5p	hsa-miR-3653-3p
hsa-miR-30c-2-3p	hsa-miR-106b-3p	hsa-miR-495-3p	hsa-miR-3677-3p
hsa-miR-139-5p	hsa-miR-200a-5p	hsa-miR-193b-5p	hsa-miR-3913-5p

2 Supplementary Figures

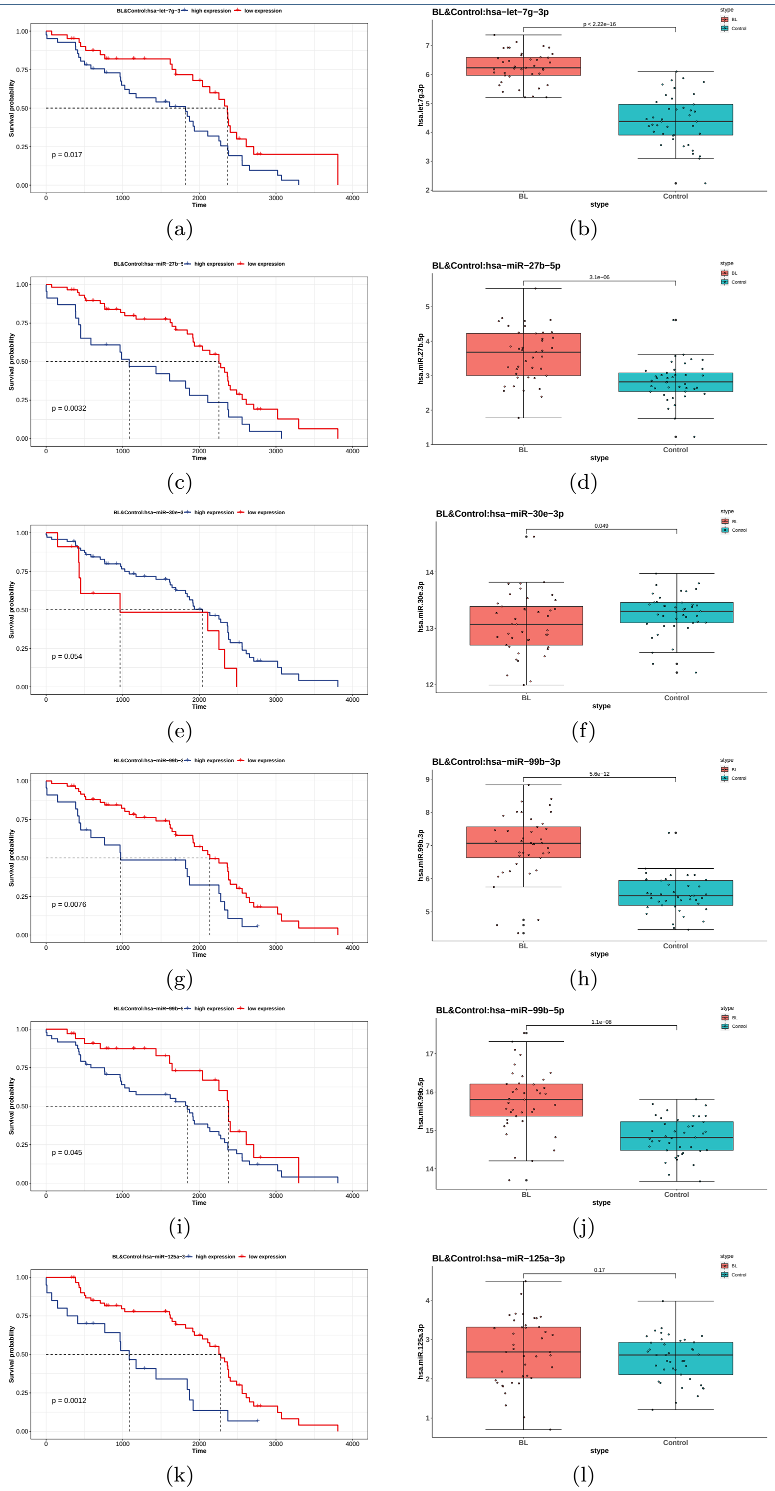

Supplementary Figure 1 Survival analysis (KM plot) and corresponding box plot of BL: (a)-(b) hsa-let-7g-3p (c)-(d) hsa-miR-27b-5p (e)-(f) hsa-miR-30e-3p (g)-(h) hsa-miR-99b-3p (i)-(j) hsa-miR-99b-5p (k)-(l) hsa-miR-125a-3p.

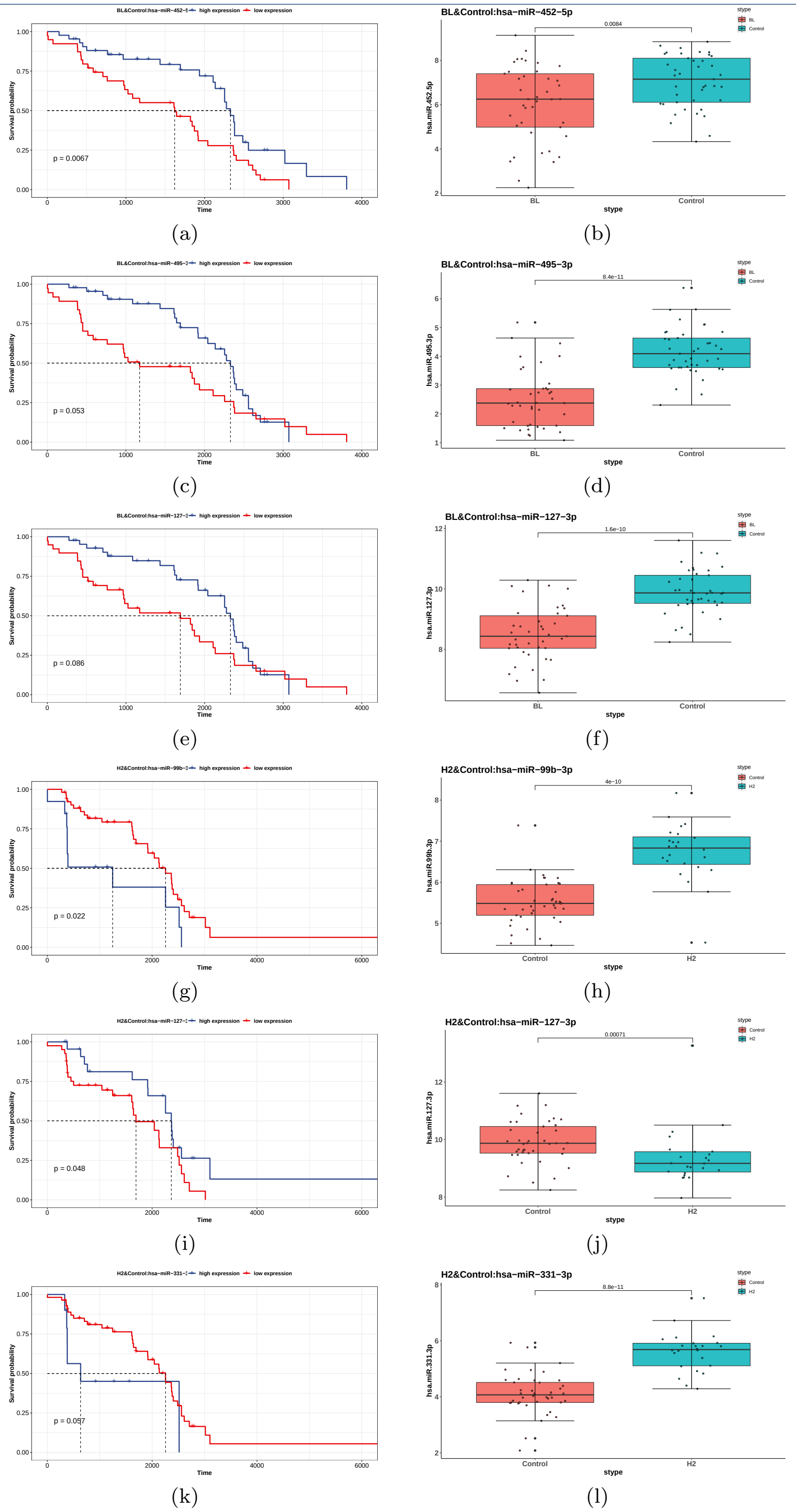

Supplementary Figure 2 Survival analysis (KM plot) and corresponding box plot of BL: (a)-(b) hsa-miR-452-5p (c)-(d) hsa-miR-495-3p (e)-(f) hsa-miR-127-3p, H2: (g)-(h) hsa-miR-99b-3p (i)-(j) hsa-miR-127-3p (k)-(l) hsa-miR-331-3p.

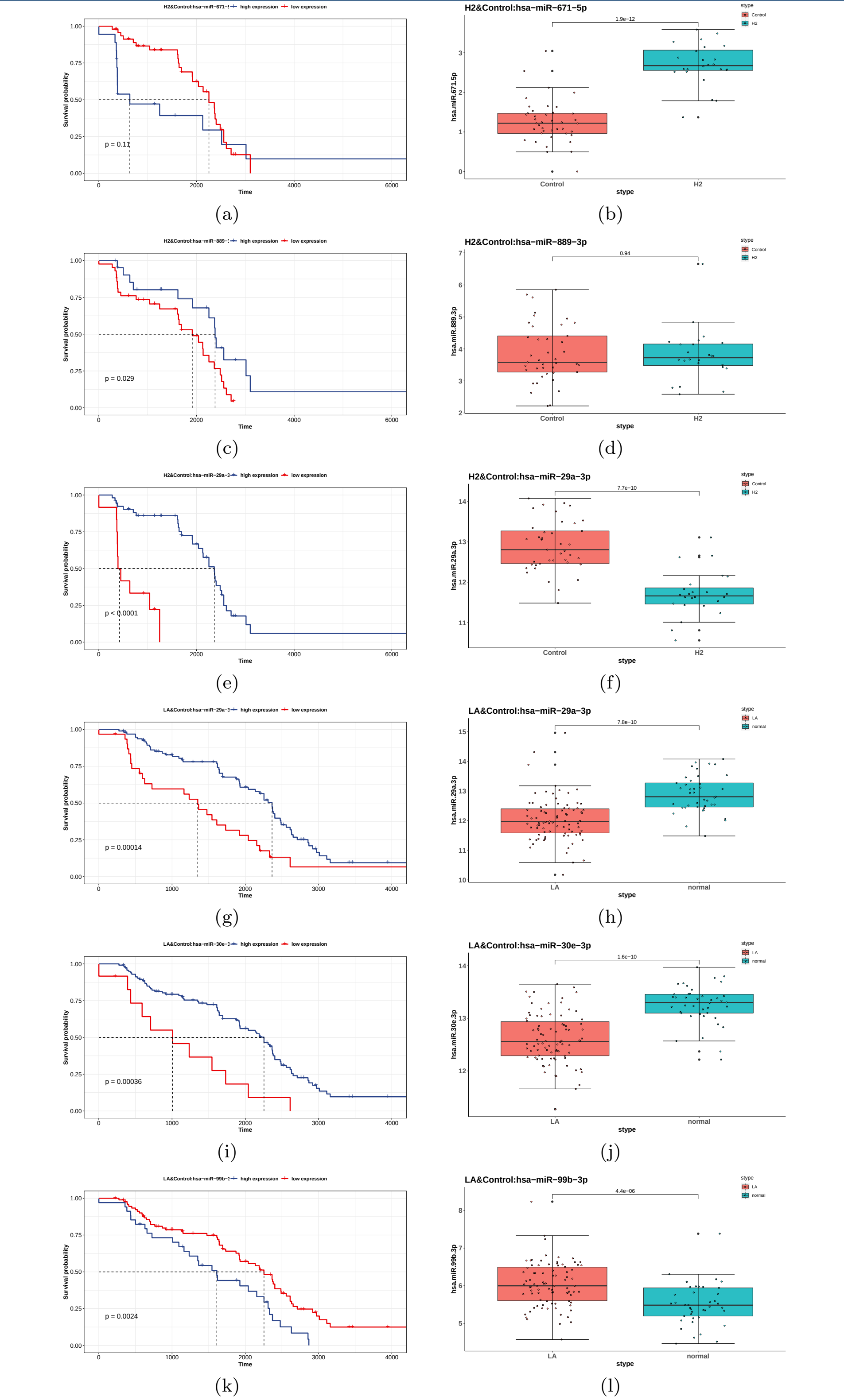

Supplementary Figure 3 Survival analysis (KM plot) and corresponding box plot of H2: (a)-(b) hsa-miR-671-5p (c)-(d) hsa-miR-889-3p (e)-(f) hsa-miR-29a-3p, LA: (g)-(h) hsa-miR-29a-3p (i)-(j) hsa-miR-30e-3p (k)-(l) hsa-miR-99b-3p.

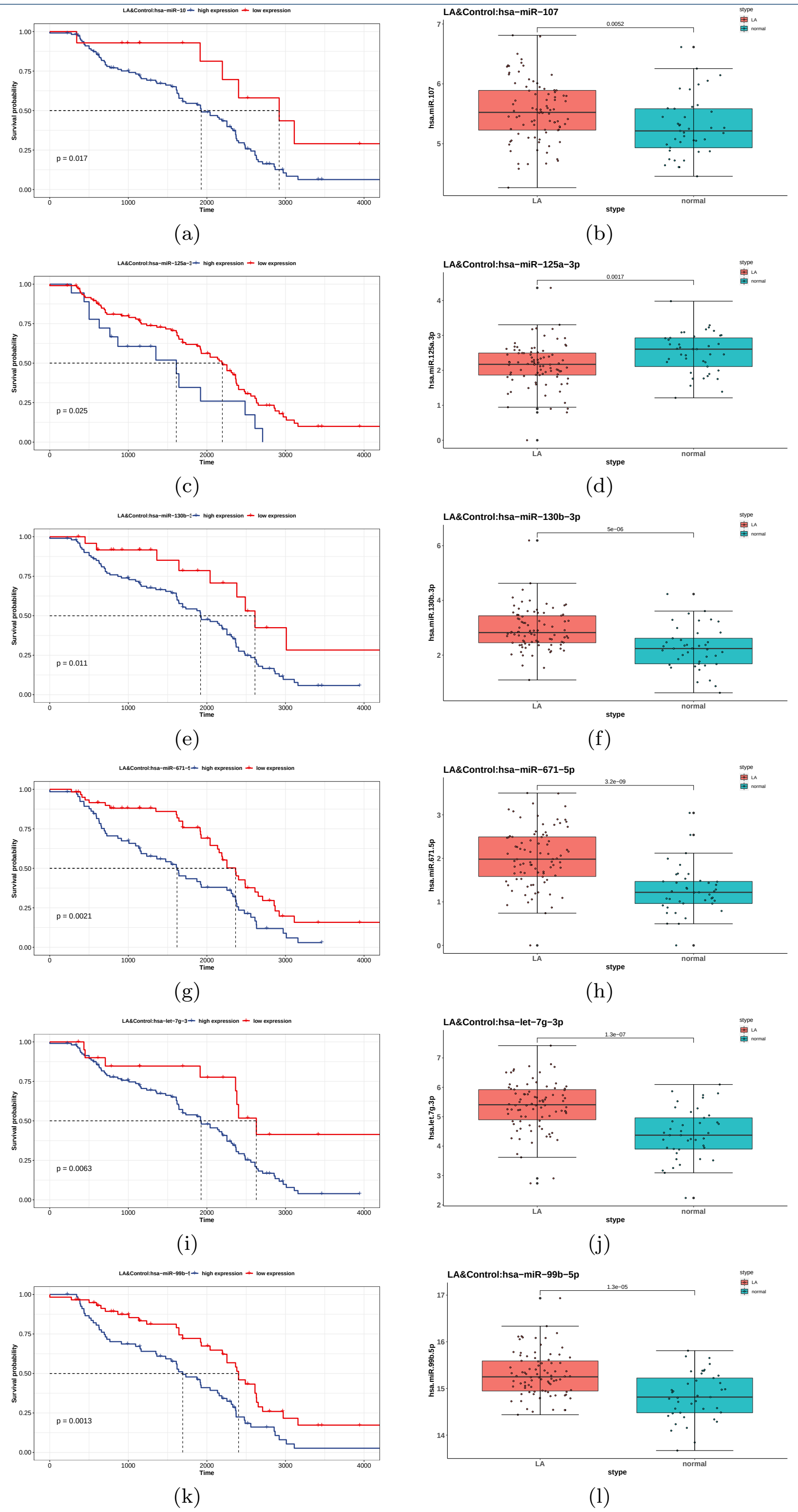

Supplementary Figure 4 Survival analysis (KM plot) and corresponding box plot of LA: (a)-(b) hsa-miR-107 (c)-(d) hsa-miR-125a-3p (e)-(f) hsa-miR-130b-3p (g)-(h) hsa-miR-671-5p (i)-(j) hsa-let-7g-3p (k)-(l) hsa-miR-99b-5p.

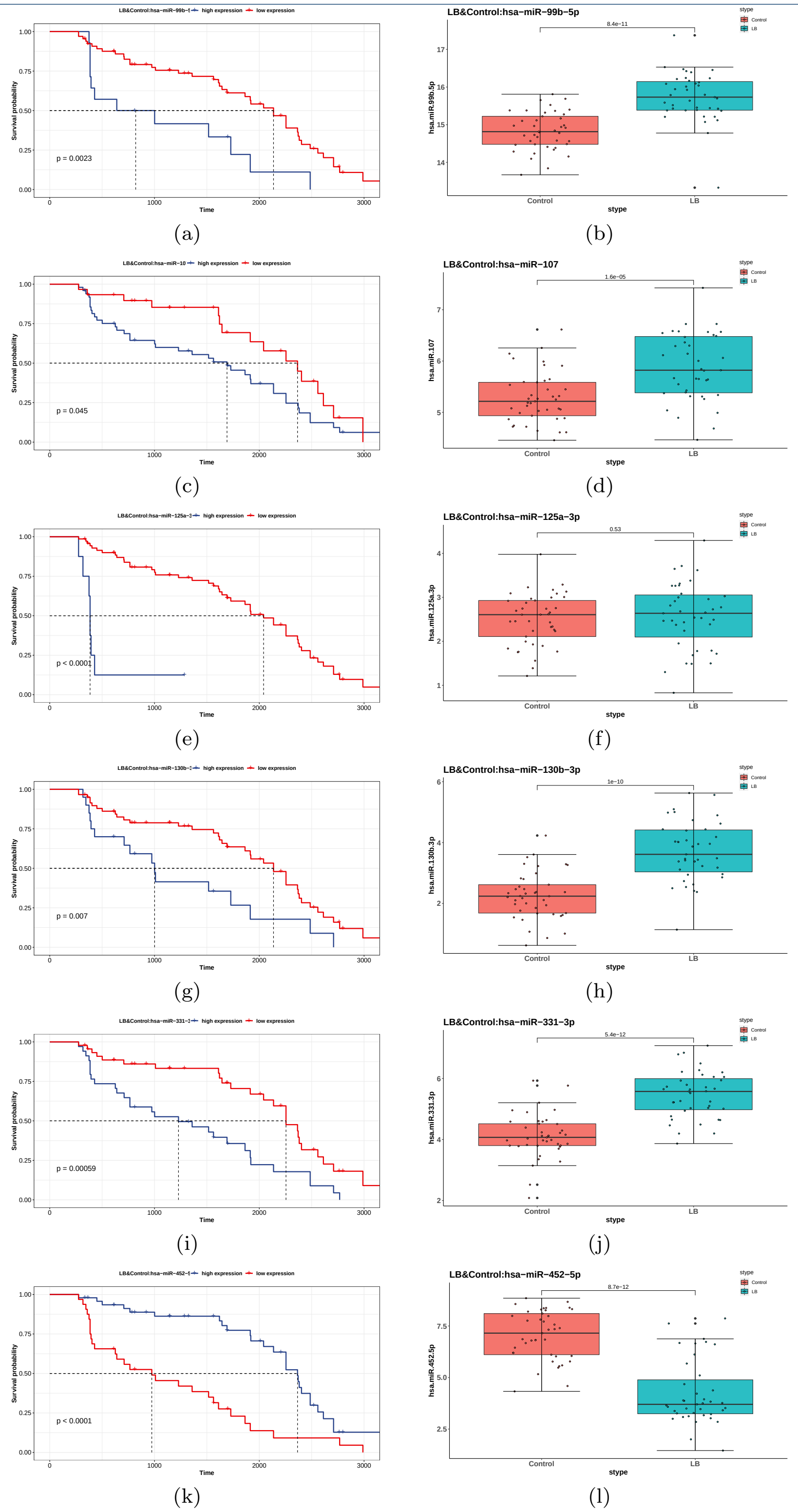

Supplementary Figure 5 Survival analysis (KM plot) and corresponding box plot of LB: (a)-(b) hsa-miR-99b-5p (c)-(d) hsa-miR-107 (e)-(f) hsa-miR-125a-3p (g)-(h) hsa-miR-130b-3p (i)-(j) hsa-miR-331-3p (k)-(l) hsa-miR-452-5p.

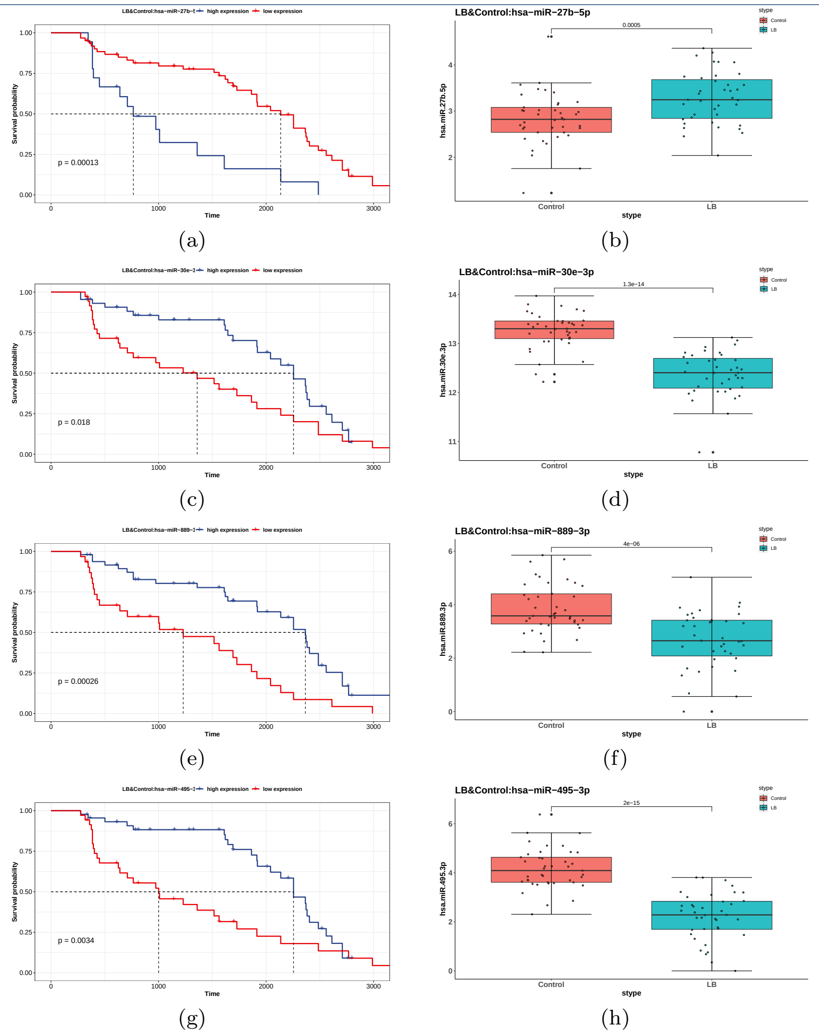

Supplementary Figure 6 Survival analysis (KM plot) and corresponding box plot of LB: (a)-(b) hsa-miR-27b-5p (c)-(d) hsa-miR-30e-3p (e)-(f) hsa-miR-889-3p (g)-(h) hsa-miR-495-3p.
